# Low complications after minimally invasive fixation of calcaneus fracture

**Published:** 2013-03-25

**Authors:** R Ene, D Popescu, C Panaitescu, G Circota, M Cirstoiu, C Cirstoiu

**Affiliations:** * Orthopaedics-Traumatology Department of Bucharest University Emergency Hospital; ** "Carol Davila" University of Medicine and Pharmacy, Bucharest

**Keywords:** osteosynthesis, complication, vascularisation

## Abstract

Calcaneus fractures are still a delicate point regarding the indication for osteosynthesis. Knowing the skin’s poor vascularisation of the back foot, the purpose of this study is to present the benefits of proper surgical options between an open and invasive osteosynthesis with anatomical reduction and internal fixation or minimally invasive approach preserving the quality of the soft parts.

66 interventions that targeted reduction and internal fixation of calcaneus fractures were performed between 2009-2012, in the Orthopaedic and Traumatology Department of Bucharest Emergency University Hospital. 29 cases underwent open reduction and internal fixation with plates and screws or Kirschner wires, and 37 cases underwent a minimally invasive reduction and Essex Lopresti osteosynthesis technique.

No patient who underwent a minimally invasive reduction had skin lesions, but showed pain due to osteoarthritis lesions that appeared in the subtalar joint. 4 of them, who underwent open reduction and internal fixation had postoperative wound infections and skin necrosis.

## Introduction

The calcaneus is the most frequently injured tarsal bone, with calcaneal fractures meaning that 60% of the fractures affect the foot and about 1% to 2% of all fractures. 75% of calcaneal fractures have an intra-articular component. Many intra-articular fractures have important long-term consequences for patients. The treatment of displaced intra-articular calcaneal fractures is still a matter of debate. There are a lot of opinions on the management of these fractures, and there have been controversies regarding the methods of treatment. 20 years ago, surgery was considered inappropriate for these fractures, and conservative treatment techniques were preferred. However, patients and surgeons remained dissatisfied with the results, and some authors have promoted surgical intervention during the last 20 years. In the past, conservative treatment was advocated following the complications of surgery and the improved results with nonoperative treatment. Starting from about 20 years ago, the unsatisfactory functional results after conservative treatment and routine computed tomography resulted in a reappraisal of the surgical approach. This change reflected the continuous dissatisfaction with the outcome of conservative treatment for these fractures, and the improvements in the surgical technique were accompanied by a reduction in complication rates. The recovery period is frequently prolonged, and a return to the pre-injury level of activity may not be reached due to pain, loss of motion, and the need for specialized footwear. However, clinical evidence supporting operative treatment for selected patient groups is limited, whereas long-term complications and adverse outcomes are still frequently documented. One of the adverse effects of the operative treatment is the damage to the soft tissues, such as flap necrosis with subsequent wound complications and sural nerve injury. To avoid these soft-tissue complications, several minimally invasive procedures have been introduced. The aim of this study was to assess the clinical, radiographic, and functional outcomes of patients, in whom displaced intra-articular calcaneal fractures were treated with a minimally invasive fixation. 

## Material and method

A comprehensive physical examination must be undertaken for each patient. One should exercise care to avoid overlooking additional injuries of the musculoskeletal system. Some patients with a fracture of the calcaneus have a concomitant spinal injury.

**Fig. 1:**
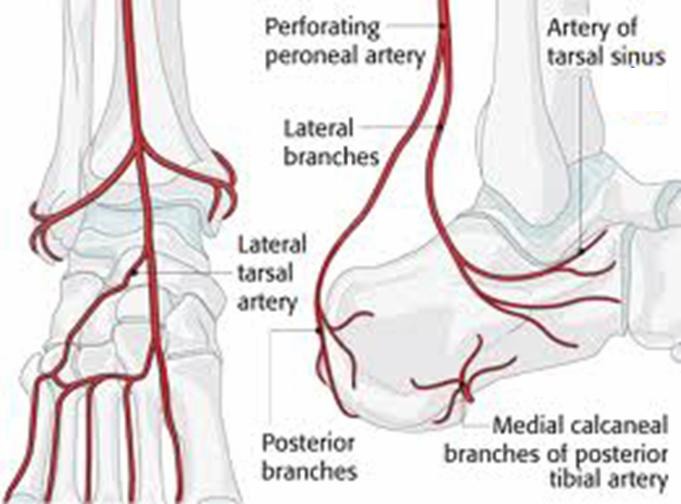
Vascularisation of the back foot

 A full history, including documentation of preexisting medical conditions such as diabetes or vascular disease, should not be unnecessarily delayed. The poor vascularisation of the back foot (**Fig. 1**) is also well known. The knowledge of the relevant anatomy is important, this way having a clear and comprehensive image of the injury. Thorough imaging allows a careful determination of the surgical approach and the planning of a staged procedure, if necessary. 

 Calcaneus fractures are still a delicate point regarding the indication for osteosynthesis. Knowing the skin’s poor vascularisation of the back foot, the purpose of this study is to present the benefits of proper surgical options between an open and invasive osteosynthesis with anatomical reduction and internal fixation or minimally invasive approach preserving the quality of the soft parts [**1**].

**Fig. 2:**
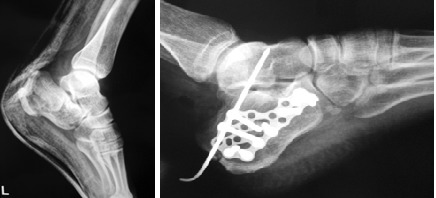
Internal fixation with plates and screws

**Fig. 3:**
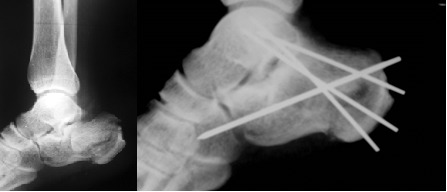
Essex Lopresti osteosynthesis technique

 66 interventions that targeted reduction and internal fixation of calcaneus fractures were performed between 2009-2012, in the Orthopaedic and Traumatology Department of Bucharest University Emergency Hospital. Patients were aged between 25-65 years and the Sex ratio M/F= 40/26. 29 cases underwent open reduction and internal fixation with plates and screws (**Fig. 2**) or Kirschner wires, and 37 cases underwent minimally invasive reduction and Essex Lopresti osteosynthesis technique (**Fig. 3**).

**Fig. 4:**
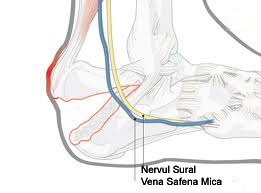
Calcaneus fractures in an extensive lateral approach

**Fig. 5:**
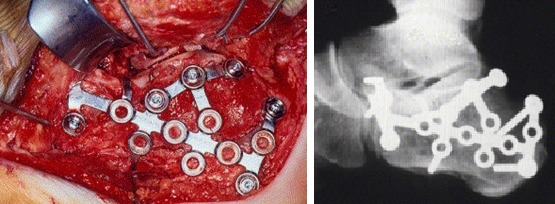
Complete reduction from the tuberosity to the anterior process and the calcaneocuboid joint

 The timing of the surgery is an important factor in the determination of the surgical success, as it is measured by long-term functional outcomes. Ideally, surgery should occur within 3 weeks after injury. This period allows for any swelling and fracture blisters to resolve completely, but the procedure is still sufficiently early to prevent premature healing and coalescence of the fracture fragments. In the absence of fracture blisters, the return of normal skin wrinkling is an indication that significant swelling has resolved, and operative intervention may proceed [**2**]. The goals of closed reduction with percutaneous fixation include the improvement of heel alignment and reduction of the posterior facet. The most popular incision for exposure during ORIF of calcaneus fractures is an extensive lateral approach (**Fig. 4**). This approach allows the surgeon to visualize the entire fracture. It also allows a complete reduction from the tuberosity to the anterior process and the calcaneocuboid joint (**Fig. 5**). In addition, this approach permits an indirect reduction of the medial wall and the sustentaculum.

The extensive lateral approach should include a full-thickness skin flap. Gentle tissue handling is necessary, also taking care of the wound closure, which is very important. Flap closure that avoids excessive tension on the skin is critical in preventing skin necrosis [**15**]. The investigations that we were obliged to use for the patients who were included in our study were the following: 

 X-rays. Represents the most common and widely available diagnostic imaging technique that creates images of dense structures, like bone, so, they are particularly useful in showing fractures. 

 Computed tomography (CT) scan. After reviewing the X-rays, the surgeon should recommend a CT scan of the foot. This imaging tool combines X-rays with computer technology to produce a more detailed, cross-sectional image of the calcaneal body. It can provide the surgeon with valuable information about the severity of the fracture. Studying CT scans helps in planning the treatment [**11**].

## Results

**Fig. 6:**
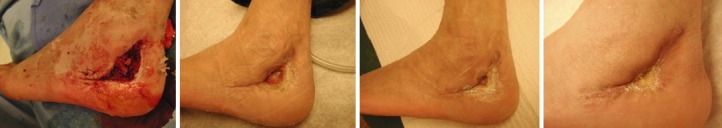
The development of a skin necrosis that delays the healing process

Postoperatively, no patients stepped on for 6 weeks, then, those who underwent open reduction and fixation with plate and screws loaded gradually, reaching full load after 10 weeks postoperatively. Postoperative recovery was excellent in 30 cases operated by using a minimally invasive approach and 20 cases underwent open reduction. No patient who underwent a minimally invasive reduction developed any skin lesions, but showed pain due to osteoarthritis lesions appearance in the subtalar joint. Among the patients who underwent an open reduction and internal fixation, 4 of them had postoperative wound infections, and 2 of them developed a skin necrosis that delayed the healing process (**Fig. 6**).

## Discussion

Intra-articular calcaneus fractures are common injuries but their treatment remains challenging and controversial. Most recent attitude favors operative treatment for most of the patients. Operative outcomes are influenced by patient selection, type of injury, anatomic reconstruction of fracture, careful management of soft tissues, and fracture institutional load [**14**]. Fracture pattern also has an influence on outcomes, with type II and III fractures in the Bohler classification [**3**] having better outcomes with surgical treatments. For comminuted fractures, there is still discussion on whether a surgical approach is warranted. 

## Conclusions

Getting the aim of an accurate reduction of the articular surface of the calcaneus, avoiding invasion of soft tissues and joints, we consider that minimally invasive osteosynthesis is the method of choice in calcaneus fractures, with a better postoperative recovery and a less rate of complications, with absolutely favorable long-term results.

## References

[1] Sanders  RW, Clare  MP (2006). Fractures of the calcaneus. In: Bucholz RW, Heckman JD, Court-Brown CM, Rockwood and Green’s Fractures in Adults, 6th ed.

[2] Sanders  R, Hansen  S (1992). Fractures of the calcaneus. In: Jahhs M, ed. Disorders of the Foot and Ankle: Medical & Surgical Management, 2nd ed,.

[3] Lowery  RBW, Calhoun  JH (1996). Fractures of the calcaneus, I: anatomy, injury mechanism, and classification. Foot Ankle Int.

[4] Palmer  I (1948). The mechanism and treatment of fractures of the calcaneus. J Bone Joint Surg Am.

[5] Bèzes H, Massart  P (1993). The operative treatment of intraarticular calcaneal fractures. Indications, technique and results in 237 cases. Clin Orthop Relat Res.

[6] Harvey  EJ, Grujic  L (2001). Morbidity associated with ORIF of intra-articular calcaneus fractures using a lateral approach. Foot Ankle Int.

[7] Paley  D, Hall  H (1993). Intra-articular fractures of the calcaneus: A critical analysis of results and prognostic factors. J Bone Joint Surg Am.

[8] Stiegelmar  R, McKee  MD (2001). Outcome of foot injuries in multiply injured patients. Orthop Clin North Am.

[9]  Buckley  RE, Meek  RN (1992). Comparison of open versus closed reduction of intraarticular calcaneal fractures: a matched cohort in workmen. J Orthop Trauma.

[10] Kundel  K, Funk  E (1996). Calcaneal fractures: operative versus non operative treatment. J Trauma.

[11] Sanders  R (1992). Intra-articular fractures of the calcaneus: present state of the art. J Orthop Trauma.

[12] Csizy  M, Buckley  R (2003). Displaced intra-articular calcaneal fractures: variables predicting late subtalar fusion. J Orthop Trauma.

[13] Sanders  R, Fortin  P (1993). Operative treatment in 120 displaced intra-articular calcaneal fractures. Clin Orthop Relat Res.

[14] Randle  JA, Kreder  HJ (2000). Should calcaneal fractures be treated surgically? A meta-analysis. Clin Orthop Relat Res.

[15] Al-Mudhaffar M, Prasad  CV (2000). Wound complications following operative fixation of calcaneal fractures. Injury.

